# Assessment of an intensive education program for pharmacists on treatment of tobacco use disorder using an objective structured clinical examination: a randomized controlled trial

**DOI:** 10.1186/s12909-022-03331-9

**Published:** 2022-04-18

**Authors:** Maguy Saffouh El Hajj, Ahmed Awaisu, Mohamad Haniki Nik Mohamed, Rana Ahmed Saleh, Noora Mohammed Al Hamad, Nadir Kheir, Ziyad R. Mahfoud

**Affiliations:** 1 College of Pharmacy, QU Health, Qatar University, 2713 Doha, Qatar; 2 Kulliyyah of Pharmacy, International Islamic University Malaysia, 25200 Kuantan, Pahang, Malaysia; 3 Sidra Medicine, Education City, Al Rayyan Municipality, Qatar; 4 College of Pharmacy and Health Sciences, Ajman University, Ajman, UAE; 5 Medical Education, Weill Cornell Medicine-Qatar, P.O. Box 24144, Doha, Qatar; 6 Division of Epidemiology, Department of Population Health Sciences, Weill Cornell Medicine, New York, NY USA

**Keywords:** Qatar, Education program, Tobacco control, Smoking cessation, Pharmacist, OSCE

## Abstract

**Background:**

Tobacco use is one of the major public health threats globally. Community pharmacists are uniquely positioned to offer tobacco cessation services owing to their easy accessibility by the public. To prepare Qatar community pharmacists to develop the competencies and skills required to offer smoking cessation services, an intensive tobacco control education program was designed and implemented. The study aimed to assess the impact of the tobacco education program on the pharmacists’ skills and competence.

**Methods:**

A random sample of community pharmacists in Qatar was chosen for participation in the program. Consenting participants were randomly assigned to either intervention or control groups. The intervention group received an intensive education program on treatment of tobacco-use disorder, while a short didactic session on a non-tobacco-related topic was delivered to the control group. The pharmacists’ tobacco cessation skills and competencies were assessed using an Objective Structured Clinical Examination (OSCE).

**Results:**

A total of 54 and 32 community pharmacists in the intervention group and the control group, respectively, completed the OSCE. The intensive tobacco education group achieved significantly higher total scores than the control group in all the OSCE cases. Specifically, the mean total scores for the intervention group were 15.2, 15.3, 14.2, 14.6, 16.3, and 15.2 compared to 8.8, 6.2, 7.7, 9.2, 8.3, and 11.3 for the control group (*p* < 0.001) for cases one to six respectively.

**Conclusion:**

The study demonstrated that an intensive tobacco cessation education program can improve pharmacists’ tobacco cessation skills and increase their tobacco cessation counseling abilities.

**Trial registration:**

Clinical Trials NCT03518476 (https://clinicaltrials.gov/ct2/show/NCT03518476**)** Registration date: May 8, 2018.

## Background

Around 942 million men and 175 million women aged 15 or older currently smoke cigarettes globally [1]. About three-quarters of male daily smokers reside in countries with a medium or high human development index (HDI), while half of female daily smokers live in very high-HDI countries [1]. In Qatar, the prevalence of tobacco smoking among adults is reported as 18.2% [2]. Despite that fewer men smoke typically in Qatar than on average in very high-HDI countries, there are over 260,000 men who smoke cigarettes daily, which makes tobacco use a public health problem in the country [2]. Hence, it is imperative for all health professionals in Qatar to offer tobacco cessation services to increase quit rates and to prevent non-smokers from starting.

Community pharmacists are uniquely positioned healthcare professionals to offer tobacco cessation services using the 5A’s (ask, advise, assess, assist and arrange follow-up) approach, owing to the ease of their accessibility by the public. Evidence has shown that quit rates achieved when pharmacists recommend smoking cessation medications and provide counseling are similar to those achieved by other healthcare professionals [3]. Additionally, community pharmacists may also provide cost-effective access to smoking cessation therapy [4]. However, a study conducted among community pharmacists in Qatar confirmed their low involvement in tobacco cessation activities. Moreover, over 80% of community pharmacists never received any formal education or training about tobacco cessation in the past [5].

Therefore, in an effort to prepare Qatar community pharmacists to have the required knowledge and skills to offer tobacco cessation services, formal tobacco education program is required. The aim of this randomized controlled study is to design, implement, and evaluate an intensive education program on tobacco-use treatment for pharmacists provided by a team of health professional educators in Qatar. The effectiveness of the program was assessed using a multiple-choice-based evaluation instrument and an Objective Structured Clinical Examination (OSCE).

OSCE is a performance-based assessment commonly used and described in the literature to assess clinical skills in health professional education programs [6–10]. Many of such programs have introduced OSCE in their undergraduate education curricula, while in some countries it is used for professional licensure purposes. An OSCE consists of a series of stations, which each examinee needs to go through in a rotational manner over a specific time. At each station, the candidate is given a simulated scenario or task, which requires performance of particular activities, such as medication history taking, medication counselling, or responding to drug information inquiry [6–10]. An OSCE station can be active (interactive or non-interactive) or inactive (e.g., rest station). Interactive stations usually involve the use of standardized patients (i.e., people who are trained to act as patients with specific medical conditions or problems, or health professionals with certain requests or tasks). A candidate at an interactive station is then assessed using a standardized rubric by an assessor. Non-interactive stations can be for preparation purposes or for the candidate to resolve a given problem without any direct observation or assessment at that point of time [6–10].

Performance of candidates being assessed via OSCE focuses on cognitive skills, including critical thinking, problem-solving, communication and interpersonal skills. In addition, elements of ethical and professional judgment can be assessed using a well-designed OSCE [6–10]. In this paper, we report the results of an OSCE used for the purpose of assessing the effectiveness of an intensive tobacco education program targeting community pharmacists in Qatar.

## Methods

### Study design

The study was a randomized controlled trial (RCT) aimed to evaluate the effectiveness of a tobacco-cessation education program for community pharmacists using an OSCE. Community pharmacists were randomly assigned to either an intensive education program on treatment of tobacco-use disorder group (intervention group) or didactic sessions on non-tobacco-related topics (control group). The tobacco dependence treatment skills of participants in both groups were assessed using a six-station OSCE, targeting evidence-based tobacco cessation core competencies. The study methodology is available in details in the published study protocol [11]. The trial is registered in Clinical Trials. NCT03518476 (https://clinicaltrials.gov/ct2/show/NCT03518476**)** with the registration date: May 8, 2018.

### Eligibility of participants and recruitment

Any licensed pharmacist who practiced in the community pharmacy setting in Qatar was eligible to participate in the study. Pharmacists in training or interns or unlicensed pharmacists were excluded.

A random sample of 529 community pharmacists was chosen for recruitment from among a total list of 1000 pharmacists, provided by the Health Practitioners Registration and Licensing Section of the Qatar Ministry of Public Health.

Consented pharmacists were randomly allocated to the intervention or control groups using permuted block randomization with blocks of size 2, 4, and 6. Randomization was made by the study statistician who was not involved in the recruitment process and was concealed from the research assistants recruiting the participants. Given the nature of the intervention, blinding of the study participants about their assigned groups was not feasible. No incentives were offered to participants in both groups other than the CE units.

### Sample size determination

The sample size estimates were for 54 participants in each group with a power of 80% to detect an effect size of 0.60 between the study groups for the numeric primary outcomes (skills scales) using the independent *t* test with a significance level of 2.5%. In addition, with 54 participants per group, the study will be able to detect a difference of at least 27.5% between the two study groups for any dichotomous outcome using the chi-square test with 80% power and a significance level of 5%. With a 10% loss-to-follow-up rate assumed, 60 pharmacists were required per group, resulting in 120 pharmacists to be randomized into groups.

### Intervention group

A team of educators, researchers, and clinicians with expertise in tobacco control and tobacco dependence treatment delivered an intensive tobacco education program to participants in the intervention group. The training program was conducted at Qatar University over four days with an average of eight contact hours per day (i.e., approximately 32 contact hours). The program materials were developed de novo and targeted the Core Competencies for Evidence-based Treatment of Tobacco Dependence by the Association for the Treatment of Tobacco Use and Dependence (ATTUD) [12]. The training program was accredited with 26.5 CE units by the Qatar Council for Healthcare Practitioners (QCHP) of Qatar Ministry of Public Health.

The training curriculum covered the following topics: (1) Tobacco-use epidemiology in Qatar and globally, (2) Risks and consequences of tobacco use, (3) Benefits of quitting tobacco use, (4) principles of nicotine addiction, 5) Non-pharmacological treatment of tobacco use and dependence 6) Pharmacological treatment of tobacco use and dependence 7) Alternative therapies 8) Counseling and communication skills 9) Relapse prevention 10) Treatment of tobacco use and dependence in special populations 11) Treatment of waterpipe dependence 12) Role of pharmacist in tobacco cessation 13) Establishing a tobacco cessation service 14) Tobacco cessation related professional development 15) Qatar law and ethics. The learning outcomes for each topic were mapped against ATTUD Core Competencies.

The program was delivered through a combination of didactic lectures and active learning strategies including problem-based learning (PBL) exercises using case scenarios, group discussions, games, role plays, videos, simulated practical applications with peers and standardized patients, self and peer debriefing and performance feedback activities.

### Control group

A didactic lecture on women health and contraception was delivered to pharmacists in the control group. This was to prevent contaminating participants’ knowledge and skills with tobacco-related elements that could subsequently change their behavior to another level not representative of their tobacco-cessation-related “current practice” or “usual care.” Pharmacists in the control group received three CE units accredited by QCHP.

### Outcome measures

The main outcome measure was the post-intervention tobacco-cessation skills, assessed through OSCE.

### Design and structure of OSCE

A six-station OSCE, which targeted the core competencies and skills covered in the program, was completed by participants in both groups in the same location of the training program. OSCE cases were developed and validated for content and face validity, through several meetings, by a group of educators and researchers experienced in the field of tobacco cessation. The first case targeted tobacco use in a generally healthy female adult patient, the second included a pregnant woman contemplating to stop smoking, the third involved a teenager who smokes, the fourth targeted relapse prevention in a patient who recently quitted and experiencing withdrawal symptoms, the fifth case involved a smoker with cardiovascular disease, and the last case involved a smoker in the precontemplation stage of behavior change. During the OSCE, each participant was allocated 10 min for each case to interact with a standardized patient (SP) in a private counseling room. Each SP was extensively trained by the research team using a validated script for the corresponding case. Performance of participants was assessed by trained assessors (faculty members from the College of Pharmacy and advanced pharmacy practitioners) using assessment checklists designed and validated by the same group of experts. The checklists included both analytical and global assessment sections. The analytical assessment section evaluated the participants’ ability to establish rapport, gather relevant information, provide appropriate recommendations and follow-up evaluation for the patient. On the other hand, the global assessment part evaluated the participants’ communication skills in general, confidence, and body language using a 5-point rating scale. The number of items in the analytical checklist differed between one case and another. Given the complexity of cases, items were rated on two or three or four level scale (1 to 4 points). Assessors in every station were trained on how to use the checklists and were blinded regarding the participants’ groups. The standardized patient was not involved in rating or assessing the study participants.

### Statistical analysis

IBM SPSS software (IBM SPSS® for Windows, Version 24.0; IBM Corp, Armonk, NY, USA) was used for data analyses. The Consolidated Standards of Reporting Trials (CONSORT) guidelines were followed when analyzing the study data [13]. Demographic and pharmacist education and practice-related questions were summarized using means and standard deviations for numeric variables and frequency (percentage) distributions for categorical variables. These characteristics were compared between the study groups and differences using the independent t-test or the Chi-square test depending on the scale of measurement of the variable. The primary outcome analyses included comparing the between-group post-training scores on the OSCE using independent t-test and univariate linear regression. Secondary analyses included using linear regression to adjust the main analyses to any potential confounders or differences in the demographic or work-related participant’s characteristics found between the two study groups. The level of statistical significance was set at 2.5% for the total OSCE score and 5% for all other comparisons.

### Ethical considerations

The study protocol and all related instruments and forms were reviewed and granted ethical approval by the Qatar University Institutional Review Board (QU-IRB) (approval number: QU-IRB 906-E/18).

## Results

### Recruitment

**Fig. 1:**
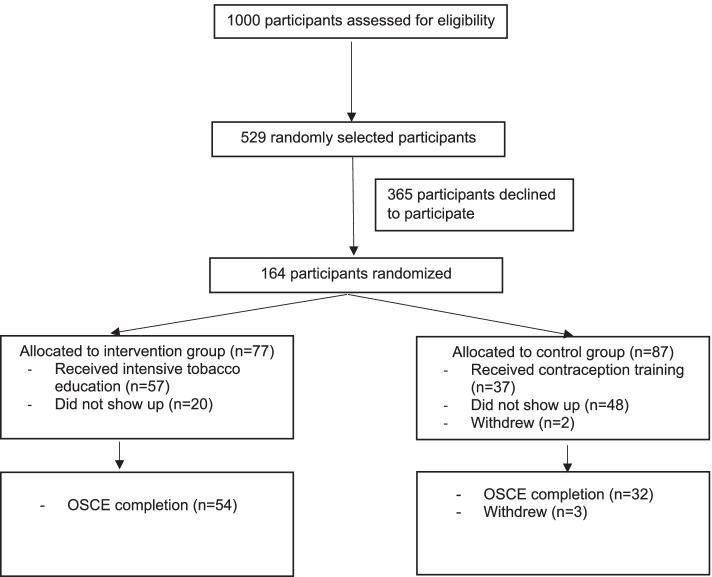
Participant flow chart

Between July and September 2018, 1000 pharmacists were assessed for eligibility and 529 pharmacists were randomly selected and invited through the phone to participate in the study. A total of 164 pharmacists accepted to participate and were randomly allocated to the intervention (*n* = 77, 46.9%) and control (*n* = 87,53.1%) groups (Fig. 1). Fifty-seven participants (74.0%) received the intensive education on tobacco dependence treatment (the intervention group), while 37 (42.5%) received the education on contraception (the control group).

Fifty-four participants in the intervention group (94.7%) and 32 participants (86.4%) in the control group completed the OSCE. The OSCE was conducted in the same week after the end of both the tobacco education and contraception training groups.

### Sociodemographic, practice and tobacco related characteristics of the participants

The majority of the participants in the intervention group were male (56.1%), while most participants in the control group were female (55.9%) (Table 1). The mean age of the participants in the intervention and the control groups was around 36 years and 35 years, respectively. The highest pharmacy academic qualification for participants in both the intervention and the control groups was B.Pharm/BSc Pharm with proportions of 85.7% and 76.4%, respectively. There were no statistically significant differences between the two groups in terms of participants’ baseline characteristics. In addition, there were no significant differences between the two groups in relation to their tobacco use status and prior training received on tobacco use management (Table 2). Majority (> 80%) of participants in both groups were non-smokers. Among the current smokers, those in the intervention group were smoking more cigarettes per day than those in the control group; 12.5 vs. 3, respectively. Almost all participants reported not having any previous training on tobacco use management.

**Table 1:** Participant socio-demographic and professional characteristics

		**Intervention group *n* = 57**	**Control group *n* = 37**	***p*-value**
		**N (%)**	**N (%)**	
**Gender**	Male	32 (56.1)	15 (44.1)	0.267
	Female	25 (43.9)	19 (55.9)	
**Mean Age (SD**^a^**)**		35.9 years (6.5)	34.9 years (6.0)	0.482
**Country of origin**	Egypt	23 (40.3)	13 (39.4)	0.276
	India	13 (22.8)	14 (42.4)	
	Palestine	0 (0)	1 (3.1)	
	Philippines	10 (17.5)	4 (12.1)	
	Sudan	5 (8.8)	1 (3)	
	Syria	1 (1.8)	0 (0)	
	Nepal	2 (3.5%)	0 (0)	
	Pakistan	3 (5.3%)	0 (0)	
**Highest pharmacy academic qualification**	B.Pharm/BSc Pha	48 (85.7)	26 (76.4)	0.606
	MPharm	5 (8.9)	4 (11.8)	
	MSc/MPhil/PharmD	2 (3.6)	2 (5.9)	
	Ph.D	1 (1.8)	2 (5.9)	
**Country from which the highest pharmacy degree was obtained**	Qatar	0 (0)	1 (2.9)	0.224
	Egypt	23 (40.3)	12 (34.2)	
	India	13 (22.8)	14 (40)	
	Jordan	1 (1.8)	1 (2.9)	
	Philippines	10 (17.5)	5 (14.2)	
	Sudan	5 (8.8)	1 (2.9)	
	Syria	1 (1.8)	0 (0)	
	Pakistan	4 (7)	0 (0)	
	Australia	0 (0)	1 (2.9)	
**Countries where the pharmacists practiced before moving to Qatar**	Egypt	23 (40.4)	14 (37.8)	0.808
	India	13 (22.8)	14 (37.8)	0.116
	Philippines	10 (17.5)	5 (13.5)	0.602
	Sudan	6 (10.5)	1 (2.7)	0.239
	Saudi Arabia	5 (8.8)	2 (5.4)	0.7
	United Arab Emirates (UAE)	1 (1.8)	1 (2.7)	1
	No previous practice	0 (0)	2 (5.4%)	0.152
	Pakistan	3 (5.3)	0 (0)	0.276
	Nepal	2 (3.5)	0 (0)	0.518
**Years of practicing as a pharmacist in Qatar**	Less than 5 years	27 (48.2)	18 (51.4)	0.724
	5–10 years	24 (42.9)	13 (37.1)	
	11–15 years	5 (8.9)	3 (8.6)	
	More than 16 years	0 (0)	1 (2.9)	
**Pharmacists’ position in the pharmacy**	Pharmacist in training	1 (1.8)	1 (2.9)	0.226
	Staff pharmacist	36 (63.2)	16 (45.7)	
	Pharmacy supervisor	9 (15.8)	5 (14.3)	
	Pharmacy manager	11 (19.3)	12 (34.2)	
	Senior pharmacist	0 (0)	1 (2.9)	

^a^Standard Deviation

**Table 2:** Tobacco-related characteristics of participants

		Intervention group	Control group	*P*-value
		N (%)	N (%)	
Smoking status	Ex-tobacco user	6 (10.7)	2 (5.9)	0.895
	Current tobacco user	3 (5.4)	2 (5.9)	
	Non-tobacco user	47 (83.9)	30 (88.2)	
Number of cigarettes per day for current smoker Mean (SD^a^)		12.5 (3.5)	3 (2.8)	0.097
Previous training on tobacco use and treatment	Yes	2 (3.8)	5 (15.6)	0.100
	No	50 (96.2)	27 (84.4)	

^a^Standard Deviation

### Post-intervention tobacco cessation skills and competencies

Table 3 summarizes the results for the OSCE cases for both intervention and control groups.

**Table 3:** Summary of OSCE^a^ results

	**Intervention group *n* = 54**	**Control group *n* = 32**	***p*-value**
	**N (%)**	**N (%)**	
**Mean (SD**^b^**) total score for Developing rapport**			
Case 1: ‘Healthy’ adult smoker Possible score range: 0–5	2.8 (0.9)	1.0 (0.7)	< 0.001*
Case 2: Pregnant smoker Possible score range: 0–5	2.7 (1.1)	0.8 (0.8)	< 0.001*
Case 3: Teenager smoker Possible score range: 0–5	2.8 (1.1)	0.5 (0.8)	< 0.001*
Case 4: Relapse prevention in a patient who recently quit smoking and has withdrawal symptoms Possible score range: 0–5	2.5 (1.0)	0.9 (0.9)	< 0.001*
Case 5: Smoker with cardiovascular diseases Possible score range: 0–5	2.6 (1.0)	1.1 (0.7)	< 0.001*
Case 6: Smoker in precontemplation stage of change Possible score range: 0–4	3.2 (1.1)	1.3 (0.8)	< 0.001*
**Mean (SD**^b^**) total score for Gathering information**			
Case 1: ‘Healthy’ adult smoker Possible score range: 0–11	5.0 (1.6)	2.8 (1.5)	< 0.001*
Case 2: Pregnant smoker Possible score range: 0–12	6.2 (2.2)	2.5 (1.6)	< 0.001*
Case 3: Teenager smoker Possible score range: 0–9	5.2 (1.6)	3.0 (1.6)	< 0.001*
Case 4: Relapse prevention in a patient who recently quit smoking and has withdrawal symptoms Possible score range: 0–13	6.0 (2.3)	4.6 (2.1)	0.005*
Case 5: Smoker with cardiovascular diseases Possible score range: 0–12	6.3 (2.1)	3.1 (1.6)	< 0.001*
Case 6: Smoker in precontemplation stage of change Possible score range: 0–11	5.0 (2.0)	4.4 (2.4)	0.184
**Mean (SD**^b^**) total score for Management Strategies**			
Case 1: ‘Healthy’ adult smoker Possible score range: 0–10	3.5 (1.7)	2.2 (1.2)	< 0.001*
Case 2: Pregnant smoker Possible score range: 0–6	2.2 (1.4)	0.4 (0.7)	< 0.001*
Case 3: Teenager smoker Possible score range: 0–7	2.1 (1.6)	1.1 (1.1)	0.002*
Case 4: Relapse prevention in a patient who recently quit smoking and has withdrawal symptoms Possible score range: 0–9	2.8 (1.3)	1.7 (1.3)	< 0.001*
Case 5: Smoker with cardiovascular diseases Possible score range: 0–10	3.0 (1.9)	1.3 (1.3)	< 0.001*
Case 6: Smoker in precontemplation stage of change Possible score range: 0–7	3.0 (1.5)	2.2 (1.7)	0.025*
**Mean (SD**^b^**) total score for Monitoring/follow-up**			
Case 1: ‘Healthy’ adult smoker Possible score range: 0–1	0.8 (0.4)	0.4 (0.5)	< 0.001*
Case 2: Pregnant smoker Possible score range: 0–2	0.8 (0.7)	0.1 (0.3)	< 0.001*
Case 3: Teenager smoker Possible score range: 0–1	0.7 (0.4)	0.2 (0.4)	< 0.001*
Case 4: Relapse prevention in a patient who recently quit smoking and has withdrawal symptoms Possible score range: -	-	-	-
Case 5: Smoker with cardiovascular diseases Possible score range: 0–1	0.7 (0.4)	0.2 (0.4)	< 0.001*
Case 6: Smoker in precontemplation stage of change Possible score range: 0–1	0.4 (0.4)	0.1 (0.3)	0.001*
**Mean (SD**^b^**) total score for global assessment**			
Case 1: ‘Healthy’ adult smoker Possible score range: 0–5	3.2 (1.4)	2.4 (1.1)	0.01*
Case 2: Pregnant smoker Possible score range: 0–5	3.6 (1.0)	2.4 (1.3)	< 0.001*
Case 3: Teenager smoker Possible score range: 0–5	3.5 (1.4)	3.0 (1.6)	0.145
Case 4: Relapse prevention in a patient who recently quit smoking and has withdrawal symptoms Possible score range: 0–5	3.4 (1.2)	2.1 (1.2)	< 0.001*
Case 5: Smoker with cardiovascular diseases Possible score range: 0–5	3.7 (1.1)	2.6 (1.6)	< 0.001*
Case 6: Smoker in precontemplation stage of change Possible score range: 0–5	3.6 (1.1)	3.3 (1.2)	0.256

^a^Objective Structured Clinical Examination

^b^Standard Deviation

#### Developing rapport scores

Pharmacists in the intervention group achieved significantly higher total scores for establishing rapport with patients than those in the control group across all the OSCE cases. For example, the mean scores for establishing rapport in the intervention group ranged from 2.5 to 3.2 compared to 0.5 to 1.3 in the control group for cases one to six.

#### Data gathering scores

Pharmacists who received the intensive tobacco-related education performed better in data gathering than those who received the contraception training. The mean scores for data gathering were significantly higher in the intervention group than the control group across all the OSCE cases except for case number six. For cases one to five, the mean scores for the intervention group ranged from 5.0 to 6.3 versus 2.5 to 4.6 in the control group.

#### Management strategies scores

With respect to management strategies, the case-specific OSCE scores ranged from 2.1 to 3.5 in the intervention group and 0.4 to 2.2 in the control group. For cases one to six, pharmacists in the intervention group achieved significantly higher total scores than did those in the control group.

#### Follow up/monitoring scores

Pharmacists in the intervention group offered appropriate follow-up/monitoring plans with significantly higher mean scores than those in the control group. The mean scores range for follow-up for the intervention group were 0.4 to 0.8 compared to the control group range of mean scores of 0.1–0.4 for cases one, two, three, five and six. There was no follow-up/monitoring section for case four.

#### Global assessment scores per case

In addition, the global assessment scores were significantly higher for the intervention group than the control group in all the cases except cases three and six. The mean global assessment scores for cases one, two, four and five ranged from 3.2 to 3.7 versus 2.1 to 2.6  for the intervention group compared to the control group.

#### Total analytical and total OSCE scores

Total analytical scores (i.e. combined scores for establishing rapport, data gathering, management strategies, and follow-up) were significantly greater in the group of pharmacists who received the intensive tobacco-related education in all the OSCE cases (Table 4).

**Table 4:** Adjusted and unadjusted total and analytical OSCE^†^ results

	**Smoking training group**		**Contraception training group**		**Unadjusted mean difference**				**Adjusted** ^**a**^ ** mean difference**			
	Mean	Standard Deviation	Mean	Standard Deviation	Mean	Standard Error	*p*-value	R^2^	Mean	Standard Error	*p*-value	R^2^—adjusted
**Case 1: ‘Healthy’ adult smoker**												
Total Analytical Score (# of items:26)	12.1	3.0	6.4	2.4	5.7	0.6	< 0.001*	49.7%	6.3	0.60	< 0.001*	63.3%
Final Score	15.2	4.0	8.8	3.1	6.4	0.8	< 0.001*	41.8%	7.2	0.80	< 0.00*	59.2%
**Case 2: Pregnant smoker**												
Total Analytical Score (# of items:24)	11.8	3.8	3.8	2.2	8.0	0.7	< 0.001*	58.9%	8.4	0.84	< 0.001*	59.7%
Final Score	15.3	4.5	6.2	3.0	9.1	0.9	< 0.001*	55.3%	9.6	1.01	< 0.001*	58.1%
**Case 3: Smoking teenager**												
Total Analytical Score (# of items:20)	10.7	3.4	4.7	2.3	6.0	0.7	< 0.001*	48.7%	6.3	0.75	< 0.001*	50.7%
Final Score	14.2	4.3	7.7	3.4	6.5	0.9	< 0.001*	39.1%	7.1	0.98	< 0.001*	43.0%
**Case 4: Relapse prevention in a patient who recently quit smoking and has withdrawal symptoms**												
Total Analytical Score (# of items:26)	11.2	3.4	7.1	3.4	4.1	0.8	< 0.001*	26.1%	3.6	0.86	< 0.001*	19.6%
Final Score	14.6	4.2	9.2	4.4	5.4	1.0	< 0.001*	27.7%	4.9	1.10	< 0.001*	21.7%
**Case 5: Smoker with cardiovascular diseases**												
Total Analytical Score (# of items:27)	12.6	3.9	5.7	2.7	7.0	0.8	< 0.001*	48.6%	6.9	0.83	< 0.001*	53.1%
Final Score	16.3	4.7	8.3	3.6	8.0	1.0	< 0.001*	45.4%	8.2	1.05	< 0.001*	50.1%
**Case 6: Smoker in precontemplation stage of change**												
Total Analytical Score (# of items:23)	11.7	3.6	8.1	4.0	3.6	0.9	< 0.001*	18.5%	4.3	0.90	< 0.001*	28.4%
Final Score	15.2	4.2	11.3	4.9	3.9	1.0	< 0.001*	15.7%	4.5	1.05	< 0.001*	28.8%

^a^Adjusted for age, gender, years of practicing as pharmacist in Qatar, smoking status and previous training in relation to tobacco use and treatment * statistically significant

The intensive tobacco education group achieved significantly higher overall total score than the control group for all the OSCE cases. Specifically, the mean total scores for the intervention group ranged from 14.2 to 16.3 compared to 6.2 to 11.3 for the control group for cases one to six (Table 4).

After adjustment for potential confounders: age, gender, years of practicing as pharmacist in Qatar, smoking status and previous training in relation to tobacco use and treatment, the intensive tobacco education group achieved, on average, significantly higher adjusted overall total analytical and total OSCE scores than the control group for all the OSCE cases. (Table 4).

## Discussion

To our knowledge, this is the first randomized controlled trial (RCT) conducted in Qatar and the Middle East that involved designing, implementing, and evaluating an intensive education program on tobacco use and dependence disorder treatment for community pharmacists using an Objective Structured Clinical Examination (OSCE). Overall, the outcomes of this study showed that an intensive tobacco education program significantly enhances community pharmacists’ skills and competencies on tobacco cessation. Generally, the pharmacists who received the tobacco cessation training achieved higher scores in developing rapport, data gathering, disease management, and patient follow up/monitoring compared to those who did not. These results are comparable to the findings of other studies, which involved pharmacists or pharmacy students elsewhere [14, 15].

The current study demonstrated the feasibility and the benefits of implementing an educational training on tobacco cessation for community pharmacists in Qatar. Evidence supports the real-life benefits of smoking cessation-training programs on pharmacists’ ability to offer smoking cessation [16–18]. One study showed that pharmacists are more likely to counsel patients on stopping smoking and to offer advice on the appropriate use of smoking cessation products when they are provided with training on smoking cessation [16]. In addition, a RCT indicated that smoking cessation rates and the possibility to quit smoking were significantly higher for patients who were counseled by trained pharmacists compared to those counseled by non-trained pharmacists [17]. Given the accessibility of community pharmacists to the general population and since most community pharmacists in Qatar are interested in offering smoking cessation counseling, [5] extensive training programs on tobacco cessation should be part of a continuous professional development (CPD) for pharmacists in Qatar. These programs can equip pharmacists with the needed skills and knowledge to overcome the burden of tobacco use in Qatar.

Different interventions and training programs related to tobacco cessation have been implemented and assessed for effectiveness among healthcare students and professionals using OSCE [19–26]. For instance, OSCE was utilized in Denver and Minneapolis, USA to assess the performance of primary care clinicians who were randomized to receive either moderate or high intensity training for motivational interviewing (MI) to address tobacco use [19]. Clinicians in the high intensity training had significantly higher scores during the OSCE as compared to those in the moderate intensity group for three of six global Motivational Interviewing Treatment Integrity scale scores [19]. The practical skills of third year medical students at a German medical school, who were randomly allocated to either an online course or an attendance course on smoking cessation, were also measured through an OSCE [20]. Overall, median OSCE scores were higher in the attendance group (70.8% vs. 62.8%; *p* = 0.087), but a statistical significance was only found in one single counselling sequence ("Assist": 66.7% vs. 51.4%; *p* = 0.049) [20]. A prospective intervention study evaluated the effects of a multimodal and interactive teaching module on smoking cessation for medical students in Germany on their knowledge, skills, attitudes, and self-reported practice using written examinations and OSCE. Scores in the OSCE were significantly higher in the intervention than those in the control group (71.5% vs. 60.5%; *p* < 0.001) [24]. Moreover, OSCE utilizing standardized patients was used for assessing tobacco dependence education of first-year dental students in one U.S. dental school. The investigators concluded that preparing for and participating in the OSCE appeared to contribute to an increase in student tobacco-related knowledge and facilitated their learning [25]. Moreover, another study implemented a teaching intervention on smoking cessation for fourth-year dental students in Germany and assessed its effectiveness on knowledge, communication skills and attitudes using written examinations and OSCE. Students in the intervention group had higher scores in OSCE as compared to the control group (74.9% vs 44.7%; *p* < 0.001; d = 2.3) [26]. The results of these programs are promising; however, extrapolating these findings to the Qatari context is not plausible especially that community pharmacy practice is different and unique in Qatar. Hence, designing, implementing and assessing the effectiveness of an intensive tobacco cessation education program targeting Qatar community pharmacists’ tobacco cessation skills using OSCE was warranted. The strengths of our program are that the program was designed and aligned according to the Core Competencies for Evidence-based Treatment of Tobacco Dependence by the Association for the Treatment of Tobacco use and Dependence (ATTUD) and the executed OSCE covered the improvement of knowledge and skills of pharmacists across six different tobacco-related case scenarios directed to patients with different levels of readiness to quit. Another strength of the study as compared to the previous studies was the prospective setup of the study and the inclusion of a control group taking the same assessments.

It is worthwhile to note that no significant improvement was observed for the intervention group for the last OSCE case, which is about a patient who was not motivated in quitting smoking (i.e., in the pre-contemplation stage of the transtheoretical model of behavior change). Initiating discussions and establishing rapport with unmotivated smokers is essential for assisting patients to quit. Hence, future training efforts should emphasize more on how to deal with these patients and encourage them to stop smoking. This might be achieved by having more practical cases and simulated scenarios that target unmotivated patients in the training program.

It is noteworthy to mention that the estimated sample size was achieved for the intervention group, but not for the control group. A possible reason for the low enrollment rate in the control group could be the topic itself. Pharmacists may not have been interested in the “women health and contraception” topic versus the “ tobacco cessation” topic.

This study has some limitations. First, allocation concealment or blinding of participants was not possible due to the nature of the study design and program. This might have resulted in some bias as the intervention group might have better prepared for the OSCE. Second, the skills of the participants in both groups were not assessed at baseline before receiving the training programs. Third, the predetermined sample size was not achieved especially in the control group due to difficulties in recruiting participants for both the training and the OSCE as most community pharmacists in Qatar do not have flexible working schedules and many have very long working hours. Moreover, in this study we were not able to examine if the study results represent real differences in the pharmacists’ tobacco cessation activities in practice. Despite these limitations, this study showed the usefulness of an intensive tobacco cessation educational program on the skills of community pharmacists. Future studies are required to examine if pharmacists’ OSCE performances are translated into real-life smoking cessation counseling interventions in their practice settings. This can be assessed through actual unannounced visits to community pharmacies using standardized patients or simulated clients to evaluate if there are any changes in pharmacists’ actual competencies in their clinical settings. Moreover, additional research is warranted to assess the impact of the program on smokers’ quitting rates in Qatar.

## Conclusion

This study has demonstrated that an intensive tobacco cessation education program can improve pharmacists’ objectively measured tobacco cessation skills and increase their abilities of tobacco cessation counseling. Any improvements in pharmacists’ tobacco cessation related skills could ultimately decrease tobacco use and dependence among their patients. We believe that the tobacco cessation program that we designed and implemented can be incorporated into a wider and larger scale CPD program in Qatar to develop a critical mass of knowledgeable pharmacists with effective tobacco cessation intervention skills.

## Data Availability

The raw data and anonymized/pseudonymized data are available on request from the corresponding author, if required. The data are not publicly available due to ethical restrictions.

## References

[1] Prevalence | Tobacco Atlas. Tobacco Atlas. https://tobaccoatlas.org/topic/prevalence/#. Accessed 28 Jan 2021

[2] Qatar | Tobacco Atlas. Tobacco Atlas. https://tobaccoatlas.org/country/qatar/. Accessed 28 Jan 2021

[3] Adams AJ, Hudmon KS (2018). Pharmacist prescriptive authority for smoking cessation medications in the United States. J Am Pharm Assoc.

[4] Csikar JI, Douglas GV, Pavitt S (2016). The cost-effectiveness of smoking cessation services provided by general dental practice, general medical practice, pharmacy and NHS Stop Smoking Services in the North of England. Community Dent Oral Epidemiol.

[5] El Hajj M, Al Nakeeb R, Al-Qudah R (2012). Smoking cessation counseling in Qatar: community pharmacists’ attitudes, role perceptions and practices. Int J Clin Pharm.

[6] Khan KZ, Ramachandran S, Gaunt K (2013). The Objective Structured Clinical Examination (OSCE): AMEE Guide No. 81. Part I: an historical and theoretical perspective. Med Teach.

[7] Khan KZ, Gaunt K, Ramachandran S, Pushkar P (2013). The Objective Structured Clinical Examination (OSCE): AMEE Guide No. 81. Part II: organisation & administration. Med Teach.

[8] Patrício MF, Julião M, Fareleira F (2013). Is the OSCE a feasible tool to assess competencies in undergraduate medical education?. Med Teach.

[9] Rushforth HE (2007). Objective structured clinical examination (OSCE): review of literature and implications for nursing education. Nurse Educ Today.

[10] Croft H, Gilligan C, Rasiah R (2019). Current Trends and Opportunities for Competency Assessment in Pharmacy Education-A Literature Review. Pharmacy (Basel).

[11] El Hajj MS, Awaisu A, Kheir N (2019). Evaluation of an intensive education program on the treatment of tobacco-use disorder for pharmacists: a study protocol for a randomized controlled trial. Trials.

[12] Association for the Treatment of Tobacco use and Dependence: Core Competencies For Evidence-based Treatment of Tobacco Dependence [Internet]. 2005. [Accessed 12 May 2018]. Available from: https://attud.org/pdf/Standards.pdf.

[13] Schulz KF, Altman DG, Moher D (2010). CONSORT 2010 Statement: updated guidelines for reporting parallel group randomized trials. BMJ.

[14] Simansalam S, Brewster JM, Nik Mohamed MH (2015). Training Malaysian Pharmacy Undergraduates with Knowledge and Skills on Smoking Cessation. Am J Pharm Educ.

[15] Martin BA, Chewning BA (2011). Evaluating pharmacists’ ability to counsel on tobacco cessation using two standardized patient scenarios. Patient Educ Couns.

[16] Sinclair H, Bond C, Lennox A (1998). Training pharmacists and pharmacy assistants in the stage-of-change model of smoking cessation: a randomised controlled trial in Scotland. Tob Control.

[17] Caponnetto P, DiPiazza J, Aiello M (2017). Training pharmacists in the stage-of-change model of smoking cessation and motivational interviewing: A randomized controlled trial. Health Psychol Open.

[18] Baliunas D, Ivanova A, Tanzini E (2020). Impact of comprehensive smoking cessation training of practitioners on patients’ 6-month quit outcome. Can J Public Health.

[19] Fu SS, Roth C, Battaglia CT (2015). Training primary care clinicians in motivational interviewing: A comparison of two models. Patient Educ Couns.

[20] Lauerer E, Tiedemann E, Polak T (2021). Can smoking cessation be taught online? A prospective study comparing e-learning and role-playing in medical education. Int J Med Educ.

[21] Ockene JK, Hayes RB, Churchill LC (2016). Teaching Medical Students to Help Patients Quit Smoking: Outcomes of a 10-School Randomized Controlled Trial. J Gen Intern Med.

[22] Fernandez K, Pandve HT, Debnath DJ (2013). Use of interactive teaching methods in tobacco cessation program and examine it by using objective structured clinical exam. J Educ Health Promot.

[23] Park KY, Park HK, Hwang HS (2019). Group randomized trial of teaching tobacco-cessation counseling to senior medical students: a peer role-play module versus a standardized patient module. BMC Med Educ.

[24] Herold R, Schiekirka S, Brown J (2016). Structured Smoking Cessation Training for Medical Students: A Prospective Study. Nicotine Tob Res.

[25] Romito L, Schrader S, Zahl D (2014). Using experiential learning and OSCEs to teach and assess tobacco dependence education with first-year dental students. J Dent Educ.

[26] Vollath SE, Bobak A, Jackson S (2020). Effectiveness of an innovative and interactive smoking cessation training module for dental students: A prospective study. Eur J Dent Educ.

